# Cutting-Edge Methods for Better Understanding Cells

**DOI:** 10.3390/cells11213479

**Published:** 2022-11-03

**Authors:** Yu Xue

**Affiliations:** Department of Bioinformatics and Systems Biology, MOE Key Laboratory of Molecular Biophysics, Hubei Bioinformatics and Molecular Imaging Key Laboratory, Center for Artificial Intelligence Biology, College of Life Science and Technology, Huazhong University of Science and Technology, Wuhan 430074, China; xueyu@hust.edu.cn

Cells are microscopic yet fundamental elements of life. The in-depth analysis of the regulatory mechanisms and phenotypic dynamics in cells is the foundation of understanding human health and diseases. Thus, the development of novel, efficient, accurate and low-cost methods has been highly encouraged for analyzing cells. In this book, I am happy to present 12 prominent papers published in *Cells* from 2020 to 2021. The methods reported in these papers covered cutting-edge techniques in single-cell analysis, gene expression data analysis, biological sequence analysis, drug discovery and genetic and/or imaging analyses.

For the single-cell analysis, five papers were selected, covering single-cell morphological monitoring, the isolation of single T cells, deep-learning-based analysis of single-cell RNA-seq (scRNA-seq) data, cross-species analysis of scRNA-seq data and validation of scRNA-seq-derived results. For the ultra-high-throughput monitoring of the morphology of individual red blood cells (RBCs), Park et al. developed a holographic cytometry instrument. Coupled with machine learning algorithms, such as logistic regression, they demonstrated that morphological changes to RBCs could be accurately measured and quantified [1]. Weiss et al. carefully tested three commonly used methods for the isolation of CD3-specific T cells from human buffy coats and peripheral blood mononuclear cells (PBMCs) based on the rationale of either immune affinity chromatography or magnetic beads. They found all three methods to be highly efficient for isolating T cells, with a high viability (>92%) and purity (>98%) [2]. For the data analysis, Walbech et al. implemented a deep learning framework of autoencoders to extract informative features from scRNA-seq data, as well as for further denoising and classification. They suggested that such a method could be useful for the identification of novel cell types not included in the training dataset [3]. Additionally, Gao et al. profiled a comprehensive transcriptomic atlas of human and mouse hematopoietic stem and progenitor cells (HSPCs), with a total of 32,805 single cells. They used a number of scRNA-seq analysis tools, such as Monocle, Seurat 2 and SCENIC, to analyze the data. Through conducting a comparison, they identified conserved HSPC types, conserved cell-type expression profiles and conserved cell-type-specific regulatory elements, motifs and networks [4]. For the validation of scRNA-seq results, Zucha et al. provided a step-by-step tutorial of a reverse transcription quantitative PCR (RT-qPCR), which could also be used for efficient screening prior to scRNA-Seq experiments. This tutorial covered sample collection, reverse transcription, preamplification, quantitative PCR and the data analysis [5].

For the gene expression data analysis, Tao et al. reanalyzed 2296 gene microarray datasets in *Caenorhabditis elegans*, and identified *rps-23*, the 40S ribosomal protein S23, as a critical housekeeping gene that could be used as a potential reference for the reliable normalization and quantification of gene expression data [6]. Esmaeili et al. performed a meta-analysis of six published bulk RNA sequencing (RNA-seq) data, and implemented three types of machine learning algorithms to identify potentially key transcription factors (TFs) to be regulated using herbal compounds in cancer cells. Using this approach, they identified four TFs, including *AIP*, *VGLL4*, *TFE3* and ID1, to be differentially expressed with the treatment of genistein, an isoflavone compound found in soy products and in liver cancer HepG2 cells [7].

For the biological sequence analysis, Wahab et al. encoded DNA sequences with each of the six types of features, such as the dinucleotide composition (DNC) and trinucleotide composition (TNC). Then, they used a convolution neural network (CNN) to develop a predictor, DNC4mC-Deep, for the prediction of potential N4-methylcytosine (4mC) in two plant genomes, *Fragaria vesca* and *Rosa vhinensis*, with the area under the ROC curve (AUC) value ranging from 0.90 to 0.96 [8].

Two papers were selected for drug discovery. First, Benchoua et al. provided a comprehensive review of various types of brain cells derived from human pluripotent stem cells (PSCs), and carefully demonstrated how these cell models were greatly helpful for the discovery of therapeutic drugs for rare genetic disorders, neurodegenerative diseases and psychiatric disorders [9]. Additionally, Dang et al. encoded small molecules using simplified molecular-input line-entry systems (SMILESs) and chemical interaction features on the CYP450 group, and developed a machine-learning-based method, histamine antagonist interaction-network inference (HAINI), by testing a variety of classification algorithms, such as the decision tree, with an AUC value of 0.96 [10].

Moreover, I selected two papers concerning genetic and imaging analyses. Kondratyev et al. reviewed the cutting-edge progress achieved in genome-wide association studies (GWASs) for the identification of genetic factors to interpret complex traits. They introduced the basic concepts and strategies in GWASs, elaborated their pros and cons, and emphasized the importance of the appropriate interpretation of GWAS findings for experimental biologists [11]. Wu et al. reviewed recent progress in using machine learning methods for the analysis of imaging and genomic data of gliomas. Three important aspects, including biomarker prediction, grade classification and prognostic prediction, were summarized. Challenges in this field, such as the lack of annotated data, data class imbalances and model overfitting, were also discussed [12].

As the Editor-in-Chief of the “Cell Methods” Section of the *Cells* journal, I am glad to have chosen these representative studies, providing leading methods or methodological reviews for *Cells* readers. For the future improvement of this section, I hope to share my thoughts here. First, the Cell Methods Section highly encourages all submissions covering the development of bioimaging, computational, bioinformatics and/or artificial intelligence methods with the capacity to help study cells. For convenience, at least one application in cells should be performed to demonstrate how the novel method can advance our understanding of cells. If possible, a brief summary on the usefulness of the method in cells should be included in the cover letter, prior to submission. I believe such a procedure would facilitate the decision making by the editorial office, as well as the following external review. Additionally, biologists working on cells would also find it easier to learn the importance of the reported methods.
